# Human Seasonal Influenza Viruses in Swine Workers in Lagos, Nigeria: Consequences for Animal and Public Health

**DOI:** 10.3390/v15061219

**Published:** 2023-05-23

**Authors:** Abdul-Azeez A. Anjorin, Aurélie Sausy, Claude P. Muller, Judith M. Hübschen, Sunday A. Omilabu, Chantal J. Snoeck

**Affiliations:** 1 Department of Microbiology (Virology Research), Lagos State University, Ojo 102101, Nigeria; 2 Department of Medical Microbiology and Parasitology, College of Medicine of the University of Lagos, Idi-Araba, Lagos 12003, Nigeria; 3 Clinical and Applied Virology Group, Department of Infection and Immunity, Luxembourg Institute of Health, L-4354 Esch-sur-Alzette, Luxembourg; 4 Department of Infection and Immunity, Luxembourg Institute of Health, L-4354 Esch-sur-Alzette, Luxembourg; 5 Centre for Human and Zoonotic Virology, Central Research Laboratory, College of Medicine of the University of Lagos, Idi-Araba, Lagos 12003, Nigeria; 6 Lagos University Teaching Hospital, Idi-Araba, Lagos 102215, Nigeria

**Keywords:** influenza A virus, human–animal interface, Nigeria, one health, reverse zoonosis, seroprevalence, swine, swine workers

## Abstract

The influenza A virus has been scarcely investigated in pigs in Africa, with rare detection prior to 2009. The spread of A(H1N1)pdm09 changed the epidemiology due to frequent human-to-swine transmission and the emergence of various new reassortants. This study therefore aimed at estimating the level of circulation and characterizing influenza A viruses at the interface between swine workers, who are crucial players in the inter-species transmission of influenza A viruses, and their animals in several farms in Nigeria, a hub for pig production in Africa. This cross-sectional study showed that 24.6% (58/236) of the pig serum samples collected in 2013–2014 had anti-influenza A antibodies in the absence of vaccination programs, but none of the pig swabs (*n* = 1193) were positive according to RT-qPCR. Viral RNA was detected in 0.9% (2/229) of swine workers sampled at their place of work, and the strains were characterized as A(H1N1)pdm09 and seasonal A(H3N2). Our results highlight that more awareness of swine workers regarding the consequences of reverse zoonosis for animal and public health is warranted. Annual vaccination and the wearing of masks when experiencing influenza-like symptoms would help decrease influenza inter-species transmission, while surveillance should be adequately supported for early detection.

## 1. Introduction

The influenza A virus causes an acute and highly contagious respiratory disease. Globally, wild birds are considered as reservoirs of influenza A viruses that can occasionally be transmitted to other hosts and establish new stable lineages, for instance in human and swine [1]. In swine, the presence of α2,3-linked sialic acid receptors for avian influenza strains and of α2,6-linked sialic acid receptors for seasonal human influenza strains in the respiratory tract led to the consideration of pigs as possible “mixing vessels” for the generation of new strains with pandemic potential [2]. Swine influenza strains have also been reported to sporadically jump to humans [3]. However, the increased surveillance worldwide in swine in the aftermath of the 2009 A(H1N1) pandemic highlighted that human-to-swine transmission is more frequent than transmission in the opposite direction [4]. In particular, swine workers are central players in inter-species transmission events due to their occupational and frequent contacts with swine [5,6]. Human-to-swine transmission of A(H1N1) 2009—hereafter called A(H1N1)pdm09—virus has been detected in swine in different locations [4]. The introduction of human seasonal influenza strains in swine herds can influence the viral genetic diversity observed in swine through adaptation and genetic drift as well as reassortments [7,8]. These new viruses require thorough monitoring to ensure fitness-for-purpose of diagnostic tests, to assess their public health relevance and possibly develop new vaccines.

Influenza A viruses are endemic in almost every part of the world, but surveillance efforts are not equally distributed. For instance, influenza A viruses have seldom been characterized in Africa. In the past two decades, at least two global events have amplified concerns about influenza in Africa: the intercontinental spread of the highly pathogenic H5 avian influenza virus by migratory birds starting in 2006, followed by amplification in local poultry populations [9], and the spread of the A(H1N1)pdm09 virus. These events highlighted the need to better understand the epidemiology of influenza A viruses in their various hosts on the continent, especially in the growing pig population, to build testing capacity and prepare for future influenza pandemics. 

Nigeria is the largest pig producer in Africa, with 8 million heads in 2021 (FAOSTAT, 2023). Most of the production is concentrated in the southern states, including Lagos State. Although farm size ranges from a few free-roaming pigs reared for individual consumption to large industrialized production systems, pig production is mainly driven by smallholders. The majority of holdings are characterized by poor hygiene and management practices as well as inadequate measures for preventing or controlling infectious diseases spread. This study therefore aimed at estimating the level of circulation and characterizing influenza A viruses at the interface between swine workers and their animals in several farms in Lagos State, Nigeria.

## 2. Materials and Methods

### 2.1. Study Design

This study took place in farm settlements for private owners, government and commercial farming in 3 divisions of Lagos State, one of the main pig producing regions in Nigeria. A questionnaire to collect farm demographic and management data was administered to farmers who agreed to participate in the study and provided informed consent. Questionnaire administration and sampling took place on the farms. 

The study was conducted in accordance with the Declaration of Helsinki, and ethical approval was obtained from the Research, Ethics, Experimentation and Grant Review Committee of the College of Medicine University of Lagos, Idi-Araba (approval ref. number: CM/COM/08/VOL.XXV) and Lagos State Ministry of Health, Alausa, Ikeja (approval ref. number: LSMH 2695/Vol.II/47). 

### 2.2. Swab Sampling and Detection of Influenza A Virus RNA

A total sample of 458 (229 nasal and 229 throat) swabs were collected from swine workers (farm owners, workers and sellers) from 6 farm settlements in June–July 2013 and March 2014. A total of 607 nasal and 586 oropharyngeal swab samples (both swabs available for 570 animals) were collected from both sick and healthy pigs in 9 farms in April-May 2013 and March 2014. All nasal samples were collected by gently swabbing surfaces of the nasal mucosa of the two nares with a sterile swab. All swabs were transferred into individual sterile cryovials containing 2 mL of virus transport medium. 

For swine workers, nasal and throat swabs were pooled for each participant with equal volumes into the lysis buffer prior to RNA extraction with QIAamp viral RNA minikit (Qiagen, Venlo, The Netherlands). For pigs, nasal swabs were screened in pools of 2 to 4 samples, according to the farm of origin. The same procedure was followed for pig oral swabs. The presence of influenza A virus was assessed by using real-time RT-qPCR targeting the matrix gene as described previously [10]. Influenza A positive samples were subtyped as A(H1N1)pdm09 or seasonal H3N2 by rRT-PCRs. Viral genome sequencing was attempted using universal primer sets [11]. Amplicons were purified and sequenced as described before [10]. Consensus sequences for the strain A/Lagos/NIE14-H-098/2013 were submitted to GenBank under the accession numbers KY317941 (matrix), KY317943 (neuraminidase) and KY317942 (non-structural protein).

### 2.3. Virus Isolation

Virus isolation from the influenza A-positive swabs was attempted on Madin-Darby canine kidney (MDCK, American Type Culture Collection, ATCC CCL-34) cells in 25 cm^2^ flasks [12]. After two blind passages, RNA was extracted from cell culture supernatant and the presence of influenza A virus assessed by RT-qPCR, as described above.

### 2.4. Serum Collection and Detection of Anti-Influenza A Antibodies

A total of 236 pig serum samples were also collected from 7 farms in Lagos State in May–June 2014. Blood samples (5–10 mL) from pigs were drawn from the femoral artery after palpation of the pulse by pulling the rare leg in dorsal recumbency or at slaughter. Blood samples were stored in Giostyle sample coolers (Gio’Style, Urgnano, Italy) stocked with ice packs before being transported to the laboratory. After coagulation at room temperature, serum was separated via centrifugation at 4000 rpm for 10 min and stored at −80 °C. 

The presence of antibodies raised against the nucleocapsid protein (NP) of influenza A virus was tested via competition ELISA (AI MultiS-Screen Ab Test; Idexx, Hoofddorp, The Netherlands) following the manufacturer’s instructions. The assay was carried out using both test kit controls and in-house test controls with serum of pigs experimentally inoculated with A/swine/Ontario/33853/05 (H3N2; positive control) and field swine sera from Luxembourg that tested negative via virus neutralization against a panel of representative strains (negative control). Optical density (OD) values were measured with Infinite M200 microplate analyzer (Tecan, Mechelen, Belgium).

## 3. Results

None of the pig swabs tested positive for influenza A virus according to RT-qPCR. In total, 58/236 (24.6%) pig serum samples were positive for anti-influenza A NP antibodies. Farm owners reported that vaccination of swine against influenza A is not economically viable and thus not implemented, indicating the circulation of wild type strains. Although the number of serum samples available was limited to reliably assess influenza A seroprevalence, within-herd seroprevalence varied greatly between farms. Indeed, seropositive animals were detected in each of the 7 farms sampled (Table 1), but within-herd seroprevalence ranged from 13.8% (Farm J) to 81.8% (Farm F; Table 1).

Pooled swabs from two swine workers (2/229, 0.9%) tested positive for influenza A virus. Positivity was then confirmed by retesting nasal and throat swabs separately: only nasal swabs were positive, with Cq values of 30.5 and 36.2. One sample, identified as seasonal H3N2 via RT-qPCR, originated from a male swine worker in his twenties (Farm C). Influenza strain subtyping as H3N2 was confirmed by Sanger sequencing. The second case, a female worker (Farm F) in her forties, was infected with a A(H1N1)pdm09 strain. No sequence could be obtained, likely due to the low viral RNA amount in the sample. Virus isolation attempts failed for both positive samples.

## 4. Discussion

In agreement with our findings, the detection rates of influenza A viral RNA in swine swabs are typically low in Africa [13,14,15]. Influenza A infection in swine usually leads to an acute infection with a limited shedding phase of 7 to 10 days, resulting in a narrow window for viral RNA detection. In Africa, the vast majority of influenza strains characterized in pigs since the spread of A(H1N1)pdm09 are closely related to those circulating in humans during the same period [16,17,18,19,20,21]. This rather suggests frequent human-to-swine transmission. Molecular investigations are also corroborated by the serological findings. After 2009, serological responses were mainly directed against A(H1N1)pdm09 with some level of cross-reactivity against other strains [14,16,22,23]. Interspecies transmission may be facilitated by sub-optimal hygiene measures. Indeed, in knowledge, attitude and practice surveys in swine workers in Ghana and Burkina Faso, the majority of swine farmers (87.7%) reported working on the farm when experiencing influenza-like illness [20], and wearing surgical masks is not common practice [13,20]. This was also illustrated in our study with the detection of seasonal A(H3N2) and A(H1N1)pdm09 strains in swine workers while sampled at their place of work. 

Our findings showed that 24.6% of pigs sampled in Lagos State had past exposure to influenza A virus. Similar seroprevalences were obtained in 2012 in Nigeria, with 27.4% of pig sera having anti-influenza A neutralizing antibodies [14] or 29.4% having anti-NP binding antibodies [23]. However, neutralizing antibodies were found in only 7.2% of swine serum samples collected in 2009 prior to the wide spread of pH1N1 in Nigeria [14], while higher seroprevalence rates were recorded in cohorts of limited size. Seroprevalences were 41.1% (HI titer > 32) in sera from 2001–2002 screened by means of hemagglutination inhibition against the human seasonal A(H1N1) strain [24], 90.1% (HI titer ≥ 20) in sera from 2008 screened against the human seasonal A(H1N1) and/or A(H3N2) [25] and 66.9% in sera from 2010 tested using ELISA [26]. In light of the relatively short life span of pigs fattened for meat production coupled with the importance of human-to-swine transmission in African settings, the timing of pig sampling compared to the influenza season in human may influence the observed disparities of (sero-)prevalence rates between studies. In our study, an A(H1N1)pdm09 strain was identified in a swine worker in one farm (Farm F) and the seroprevalence in pigs was high two months after, highlighting the possibility of human-to-swine followed by swine-to-swine transmission in the herd, which a suitable sampling timeframe allowed us to monitor. Interestingly, Ayim-Akonor et al. observed a difference in the prevalence of viral RNA-positive swine swabs according to the season, with detection rates 2.5 times higher during the rainy season compared to the dry season in Ghana [20]. The rainy season also corresponds to the period of increased influenza virus transmission in the Ghanaian population [27]. Similar observations were made in Kenya, where increased seroprevalence in pigs followed periods of transmission in human population during colder months [28]. Additional variations in influenza circulation in the human population, such as unusual circulation patterns or different dominating subtypes, may also influence transmission to swine but need to be further investigated. Similarly, factors that may further promote interspecies transmission and virus circulation dynamics within farms in local settings require additional considerations.

In previous studies in Nigeria, no virus was isolated or no viral RNA was detected via RT-qPCR from swine workers from Lagos with clinical signs of influenza-like illness [26,29]. To our knowledge, this study thus provides the first evidence of the molecular detection of influenza A virus in swine-workers in Nigeria. Although the strains identified here are typical human seasonal influenza viruses and are not linked to swine husbandry, our findings reiterate that infectious swine workers tending to animals may serve as a source of viruses to their pigs. Greater awareness of swine workers regarding the reverse zoonosis of influenza A viruses is needed to improve animal health, to avoid endemic circulation in swine and possibly the generation of reassortant viruses with new genetic and antigenic properties. Annual vaccination of swine workers and wearing masks when experiencing influenza-like symptoms would constitute valuable tools to decrease the inter-species transmission of influenza A viruses, while surveillance should be adequately supported, both financially and politically, as an early detection program. 

## Figures and Tables

**Table 1:** Prevalence of anti-NP influenza A antibodies in swine sera and influenza A RNA in swine and human respiratory swabs from 17 farms in Lagos State, Nigeria.

Lagos State Division	Local Government Area	Farm	Swine Samples				Human Samples	
			No. of Sera Tested	No. of Sera Positive for Anti-NP Antibodies (%)	No. of Nasal Swabs Tested	No. of Oropharyngeal Swabs Tested	No. of Participants Tested ^d^	No. of Influenza Virus Positive Participant (%)
Ikeja	Agege	A	-	-	74 ^a^	74 ^a^	-	-
		B	-	-	-	-	13 ^e^	0 (0.0)
		C	-	-	101 ^a^	100 ^a^	99 ^e^	1 (1.0)
		D	17 ^c^	4 (23.5)	-	-	-	-
		E	137 ^c^	23 (16.8)	-	-	-	-
	Ojo	F	22 ^c^	18 (81.8)	74 ^a,b^	74 ^a,b^	46 ^b,f^	1 (2.2)
		G	7 ^c^	3 (42.9)	-	-	-	-
Badagry	Badagry	H	-	-	30 ^a^	30 ^a^	12 ^e^	0 (0.0)
		I	-	-	98 ^a^	99 ^a^	-	-
		J	29 ^c^	4 (13.8)	-	-	-	-
		K	-	-	97 ^a^	82 ^a^	47 ^f^	-
Ikorodu	Ikorodu	L	-	-	43 ^b^	39 ^b^	-	-
		M	-	-	46 ^b^	47 ^b^	-	-
		N	5 ^c^	2 (40.0)	-	-	-	-
		O	19 ^c^	4 (21.1)	-	-	-	-
		P	-	-	-	-	12 ^b^	0 (0.0)
		Q	-	-	44 ^b^	41 ^b^	-	-
Total		17	236	58 (24.6)	607 ^g^	586 ^g^	229	2 (0.9)

^a^ Sample collection in April–May 2013. ^b^ Sample collection in March 2014. ^c^ Sample collection in May–June 2014. ^d^ Both nasal and throat swabs were obtained for each participant, totaling 458 swabs. ^e^ Sample collection in June–July 2013. ^f^ Date of sample collection missing. ^g^ All swab samples, tested in pools, were negative for influenza A RNA.

## Data Availability

The data related to human samples and questionnaires are not publicly available due to ethical restrictions. The raw data that support the findings related to pigs are available from the first author upon reasonable request.
